# Acceptability of on-site rapid HIV/HBV/HCV testing and HBV vaccination among three at-risk populations in distinct community-healthcare outreach centres: the ANRS-SHS 154 CUBE study

**DOI:** 10.1186/s12879-020-05601-7

**Published:** 2020-11-16

**Authors:** Ruxandra Calin, Véronique Massari, Gilles Pialoux, Nelly Reydellet, Eve Plenel, Carole Chauvin, Marie Jauffret-Roustide, Nesrine Day, Georges Kreplak, Anaenza Freire Maresca, Nicolas Derche, Sandra Louis, Stanislas Pol, Véronique Doré, Christine Rouzioux, Pierre Chauvin

**Affiliations:** 1 Service de Maladies Infectieuses, Hôpital Tenon, Groupe Hospitalier Est, AP-HP, 4 rue de la Chine, 75970 Paris, Cedex 20 France; 2 Inserm, IPLESP, ERES, Institut Pierre-Louis d’épidémiologie et de santé publique, Sorbonne Université, 75012 Paris, France; 3 Sorbonne Université, UPMC Université, Paris 06, France; 4 Le Kiosque, Checkpoint-Paris, Groupe SOS, Paris, France; 5 Cermes3, Inserm U988, CNRS UMR8211, EHESS, Université de Paris, Paris, France; 6 Laboratoires Centre Biologique Chemin Vert (CBCV), Paris, France; 7 ARCAT, Pasaje Latino, Groupe SOS, Paris, France; 8 AP-HP, Hôpital Ambroise Pare, Service de Médecine Interne, Boulogne-Billancourt, France; 9 CSAPA 110 Les Halles, ARCAT, Groupe SOS, Paris, France; 10 AP-HP, Hôpital Cochin, Service d’hépatologie, Paris, France; 11 ANRS: Agence Nationale de Recherche sur le sida et les hépatites virales, Paris, France; 12 Université Paris Descartes, Sorbonne Paris Cité, Faculté de Médecine, Paris, France

**Keywords:** On-site rapid HIV testing, HBV testing, HCV, HBV vaccination, Linkage-to-care, Community sites

## Abstract

**Background:**

HIV, HBV and HCV infections continue to represent major health concerns, especially among key at-risk populations such as men who have sex with men (MSM), people who inject drugs (PWIDs), transgender women (TGW) and sex workers (SW). The objective of the ANRS-CUBE study was to evaluate the acceptability of a healthcare, community-based strategy offering a triple rapid HIV-HBV-HCV testing, and HBV vaccination, targeted at three priority groups (MSM, PWIDs and TGW/SWs), in three community centers, in the Paris area.

**Methods:**

This longitudinal multicentric non-randomized study included all adult volunteers attending one of the three specialized community centers in Paris, between July 2014 and December 2015. HIV, HBV and HCV status and acceptability of HBV vaccination were evaluated.

**Results:**

A total of 3662, MSM, 80 PWIDs and 72 TGW/SW were recruited in the three centers respectively. Acceptability of rapid tests was 98.5% in MSM and 14.9% in TGW/SWs, but could not be estimated in PWIDs since the number of users attending and the number of proposals were not recorded. User acceptability of HBV vaccination was weak, only 17.9% of the eligible MSM (neither vaccinated, nor infected) agreed to receive the first dose, 12.2% two doses, 5.9% had a complete vaccination. User acceptability of HBV vaccination was greater in PWIDs and TGW/SWs, but decreased for the last doses (66.7 and 53.3% respectively received a first dose, 24.4 and 26.7% a second dose and 6.7 and 0% a third dose). Fifty-three participants (49 MSM and 4 PWIDs) were discovered HIV positive, more than half with a recent infection. All but two HIV positive participants were linked to appropriate care in less than one month.

**Conclusions:**

Rapid HIV-HCV-HBV screening showed a very high level of acceptability among MSM. Efforts need to be made to improve immediate acceptability for HBV vaccination, especially among MSM, and follow-up doses compliance. Our results show the important role of community centers in reaching targets, often fragile, populations, while also suggesting the need to reinforce on-site human support in terms of testing and vaccination, especially when addressing PWIDs.

## Background

Human immunodeficiency virus (HIV), chronic hepatitis B virus (HBV) infection and chronic hepatitis C virus (HCV) infection continue to represent major global public health concerns [1, 2]. Since 2011, UNAIDS has been recommending, targeted interventions for key populations that share a greater risk for infections with regards to low access to care, particularly among men who have sex with men (MSM), sex workers (SW) and their clients, and people who inject drugs (PWIDs) [3]. Recommendations also reinforce the need for combined screening of these three infections with often overlapping epidemiological patterns [4]. In order to meet the ambitious goals by the Global Health Sector Strategy (GHSS) and UNAIDS with respect to prevention and efficient therapy, a better on-field deployment of existing testing tools addressing at-risk population, access to adapted counseling and a more efficient linkage-to-care, need to be implemented [5, 6].

In France, a recent evaluation based on newly diagnosed HIV cases from 2004 to 2014 estimated that in 2014, for an incidence of 0.17‰ (95% confidence intervals (CI): 0.16 to 0.18) or 6607 (95% CI: 6057 to 7196) newly diagnosed HIV infections, undiagnosed HIV prevalence was of 0.64‰ (95% CI: 0.57 to 0.70) or 24,197 (95% CI: 22,296 to 25,944) adult infections, with a median time to diagnosis of 3.3 years [7]. Rates of incidence and undiagnosed prevalence were higher in Paris and overseas regions (2–10-fold), born-abroad MSM (respectively, 108- and 78-fold), French-born MSM (62- and 44-fold), born-abroad PWIDs (14- and 18-fold) [7]. HIV testing activity increased by 12% between 2010 and 2017 in France, but this increase was not accompanied by an increase in the number of confirmed positive serologies, suggesting probably that screening may not have benefited the most HIV-exposed populations [8]. Moreover, despite a very light decrease in HIV French incidence of 7% in 2018 compared to 2017, one-third of HIV-positive discoveries were made at the time of an advanced HIV infection [9]. As in previous years, overseas departments and the Paris region accounted for the highest discovery rates (150–896 new HIV infections/10^6^ inhabitants) [9].

Regarding HCV infections in France, it was estimated that in 2015 approximately 75,000 individuals were unaware of their infection [10]. Injection drug use is the major route of transmission for HCV infection in high income countries, with seroprevalence studies showing in France a rate of 64% of HCV positive PWIDs, with up to 27% of them being unaware of their positive status from ANRS-Coquelicot Study [11, 12]. The same study showed that HBV prevalence (measured by HBs Ag) reached 1.4% among people who use drugs (PWUDs) and 0.8% among PWID, and the risk factors associated with HBV were being born in a moderate/high endemic zone and living in precarious conditions [13]. The ANRS-PREVAGAY study, among MSM attending gay venues in 5 French metropolitan cities, showed that chronic HCV infection prevalence was significantly higher in HIV-positive MSM (3%) and especially among MSM declaring slamming practices (injection in sexual contexts), chemsex and risky sexual behaviors [14]. The same study showed a 1.5% HBV prevalence in HIV positive MSM, compared to a national HBV prevalence in France of 0.65% [14]. SW and among them especially transgender women (TGW) and those using IV drugs are more vulnerable to HIV and HBV and/or HCV infections, with prevalence highly influenced by migration and socio-demographic patterns. In France HIV and HBV prevalence were estimated respectively at 17.2% among TG SW and 4.2% among SW (male, female or TG) [15].

Regarding HBV vaccination, National French guidelines recommend that all MSM with multiple sex partners should be vaccinated against HBV, but a substantial proportion of individuals in this population still remain unvaccinated [14, 16]. SW and PWIDs are also clearly identified as key populations for HBV vaccination.

Delocalization of screening and access to care is recommended in WHO elimination goals. Rapid tests proposed by on-site health professionals in community settings close to the target populations and identified by the latter as adapted to their needs, constitute a complementary tool to the existing offer of standard voluntary screening for HIV, HBV and HCV [17–19]. Point-of-care (POC) testing could facilitate screening by reducing the material or psychological obstacles of standard testing and provide ease in obtaining results and increased linkage-to-care, including access to antiviral therapy [17]. Several studies have showed higher acceptability and test results delivery when using rapid tests with a beneficial impact for high risk groups [17, 20–22]. An immediate vaccination offer after on-site screening might also increase the efficacy of the intervention. However, rarely do studies extend to different key populations and use combined strategies of testing simultaneously for the three viruses, associated with an HBV vaccination offer.

Thus, the objective of the ANRS-CUBE study was to evaluate the acceptability of an on-site rapid triple testing for HIV, HBV and HCV and the acceptability of a three-dose HBV vaccination proposal, among three different key populations: MSM or bisexual, PWIDs and TGW/SW attending three community centers in the Paris area.

## Methods

### Ethical statement

A French ethical committee approved the study on July 25, 2013 (Comité de Protection des Personnes d’Hôtel-Dieu, Paris, France). An information note available in French, Spanish, Russian and English, was provided to all individuals offered to participate in the ANRS-CUBE study. The study was registered with the French National Agency for Drug Safety (ANSM) on June 52,013 under the number 2013-A00840–45. In accordance with the French law all study-related computerized as reported to the Advisory Committee on the processing of research information in the field of health and the CNIL (National Commission of Informatics and Liberties).

### Study design and participants

#### Study design

The non-randomized, prospective ANRS-CUBE study recruited volunteers from three community centers in Paris dedicated to receiving, respectively, MSM, PWIDs and TGW/SW, considered as priority target populations for testing and access to health-care.

In order to increase awareness, informational posters about the study were displayed in the three centers. A 6-month period was added to evaluate linkage-to-care of participants detected positive for one (or more) of the three infections; and to follow the three-dose HBV vaccination plan in volunteers negative for HBs Ag.

Through partnership agreements, individuals screened positive were referred to different specialized health care providers (hospitals, health centers and/or general practitioners trained in HIV/HBV/HCV follow-up). This is refered to as linkage to care throughout the report. A systematic phone-call was organized in order to facilitate and optimize the access to care and the therapeutical follow-up.

**Fig. 1:**
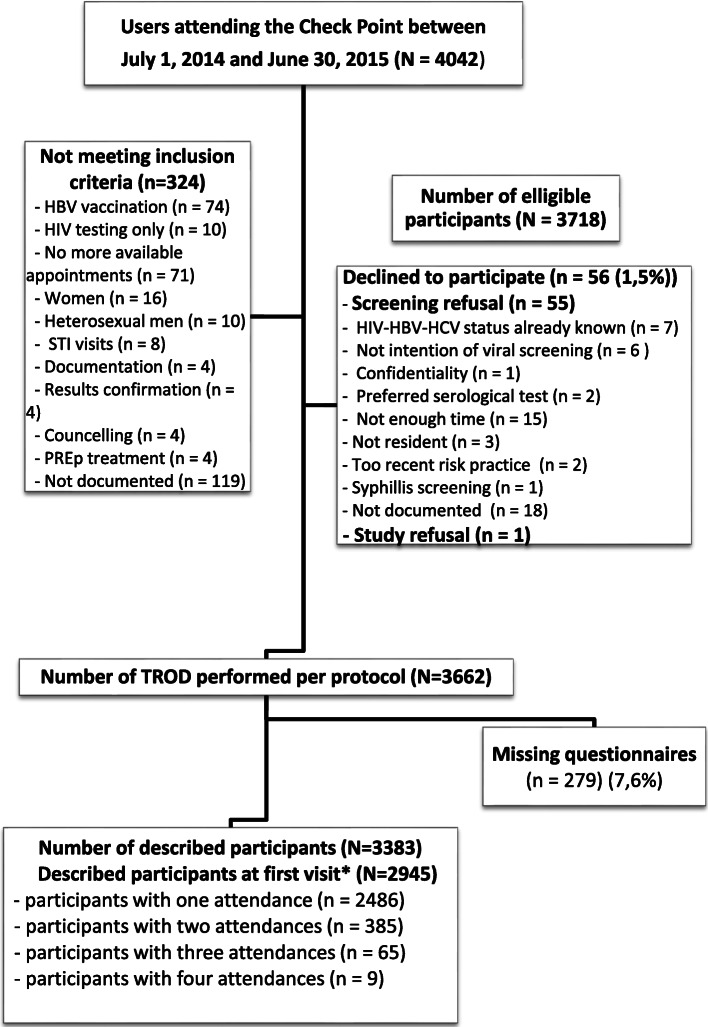
Study Flow diagram Check-point. *Only the first individual visit was included in the analytical sample

Among the 3718 individuals eligible from CP alone, 3662 were triply tested for HIV, HBV and HCV and 2945 were the number that were properly described using the Questionnaire (Fig. 1). That the same pattern was applied for the other recruiting centres.

The rapid tests used were: the INSTI® HIV-1 / HIV-2 rapid test (specificity: 99.30% with 95% CI [98.90–99.50] and sensitivity: 99.60% with 95% CI [98 (99.92%) with 95% CI [99.42–99.98] and sensitivity: 99.79% with 95% CI [98.79–99.96]); OraQuick HCV® (Specificity: 99.90% with 95% CI [99.6–100] and sensitivity: 99.7% with 95% CI [99.0–100]) and TOYO® ANTI-HCV Specificity: 98.0% with 95% CI [94.4–99.3] and sensitivity: 96.4% with 95% CI [93.3–98.1]); VIKIA HBs Ag® (specificity: 99.90% with 95% CI [99.44–100] and sensitivity: 98.33% with 95% CI [96.59–99.33]). All tests had EC marking. Results of the HIV rapid test were made available almost immediately, whereas HCV and AgHbs tests results required approximately 20 min. The HBV Engerix B20 Vaccine (GSK) was proposed to eligible participants (without a previous HBV infection) and that reported no HBV vaccination.

A recent HIV infection was defined as contamination during the last 6 months, documented by an incomplete Western blot test [23].

Study sites:

- The “Checkpoint-Paris” (CP), a community-based voluntary counselling and HIV testing site (CBVCT) localized in a gay and touristic neighborhood of central Paris receiving approximately 10,000 persons each year, mainly MSM, as part of its rapid HIV testing offer.
- The “110 Les Halles” (110 LH), CSAPA (Center of Care, Support and Prevention in Addiction), a low-threshold structure of care providing PWIDs with information, medical and psychological support and access to harm reduction tools, localized in a touristic neighborhood of central Paris and receiving about 550 persons each year, mainly PWIDs who live in highly precarious conditions with a large proportion of migrants coming from Eastern Europe and Russia.
- El Pasaje Latino (PL) (ARCAT Association), a community center entirely devoted to receiving SW and TG, providing access to social services and risk reduction materials. It is located in a deprived neighborhood in the north of Paris, receiving around 600 persons each year, mainly trans-women and men sex workers coming from South America.

#### Informed consent and questionnaires

Between July 1, 2014 and December 31, 2015, a systematic face-to-face proposal of on-site triple rapid testing for HIV, HBV and HCV was made to all adult individuals of the target groups attending one of the three recruiting centers. During the interview, the physician or the recruiting staff member read the information note to the potential participant and answered all questions. Written consent was obtained from all consenting volunteers. Questionnaires were filled-in by participants in order to evaluate feasibility and acceptability of rapid tests as well as for the three-dose HBV vaccination. Questionnaires were performed after the triple testing which explains for the variation in sample size.

In case of refusal, a questionnaire recording the reason for refusal was proposed.

### Study endpoints

The primary endpoint of the study was to assess the acceptability of rapid HIV, HBV and HCV testing in the three target populations.

The secondary endpoints were to evaluate the efficacy of the combined rapid response offer (measurement of proposal rates, acceptability and achievement), measure of linkage to care (rate of people screened with one or more positive tests successfully addressed to a specialized health care giver) and the efficacy of the HBV vaccination offer (measurement of the proposal, acceptance and implementation rates).

### Inclusion criteria

All adult (more than 18 years old) consenting individuals attending the three recruiting sites during the study period were eligible for enrolement.

### Exclusion criteria

Individuals under 18 years of age, persons under guardianship, persons unable to provide written consent or unable to undergo an interview and persons who refused their participation, were not included. Known HIV, HBV or HCV positivity was not an exlusion criteria.

### Sample size determination

Based on numbers of attendants in 2011 (more than 2500 for the CP, 551 for 110 LH and 403 for PL) and in the absence of data on the acceptance of combined tests for the three viral infections in these populations, the number of the study participants required was calculated for an acceptance rate of 65% at the CP and 20% in the other two centers: 1625 users had to be included at the CP, 110 at 110LH and 81 at PL.

### Statistical analysis

Study participants were selected by consecutive sampling and described only once, at their first visit, but acceptability rates were calculated on the total number of proposed viral tests, each proposal counting as an individual testing opportunity. Only the first individual visit was included in the analytical sample, which represents the denominator for all prevalence values. The acceptability of the testing strategy was evaluated by statistical analysis of questionnaires and of activity data in each center. Nominal variables were presented as counts and their corresponding percentages. All statistical analyses were carried out using STATA (version 13.1; StataCorp, College station, TX).

## Results

### Study baseline characteristics in the three populations and triple HIV-HBV-HCV testing acceptability

A total of 3718 and 584 participants were screened for eligibility in the CP and PL centers, respectively. Among the eligible participants who accepted testing in one of the three centers, we describe the baseline characteristics at the first study visit of 2945, 80 and 72 participants, in the CP, 110 LH and PL centers, respectively, that filled-in study questionnaires (Table 1). The sexual orientation for participants in each center, sexual risk behaviors and psychoactive substance-use in the last 12 months are described in Table 2. The history of screening and HBV vaccination is presented in Table 3.

**Table 1:** Selected participant demographics, by study site

	Checkpoint *n* = 2945	110 Les Halles *n* = 80	El Pasaje Latino *n* = 72
	N (%)	N (%)	N (%)
Gender			
Men	2907 (98.7)	71 (88.7)	28 (38.9)
Women	0 (0)	8 (10.0)	20 (27.8)
Transgender people	11 (0.4)	0 (0)	24 (22.2)
Don’t want to be defined	27 (0.9)	1 (1.2)	0 (0)
Age			
18–29 years	1392 (47.3)	12 (14.6)	16 (22.2)
30–39 years	929 (31.5)	22 (26;8)	22 (30.6)
40- and more years	624 (21.1)	48 (59.1)	33 (45.8)
Origin			
French	2397 (84.2)	38 (47.5)	4 (5.8)
European citizens	220 (7.7)	13 (16.2)	11 (15.9)
Others	230 (8.1)	29 (36.2°	54 (78.3)
Region of birth			
France	2239 (78.7)	36 (45.0)	2 (2.9)
Europe	198 (7.0)	16 (20.0°	6 (8.7)
USA and South America	142 (5.0)	2 (2.5)	51 (73.9)
Asia and Oceania	109 (3.8)	2 (2.5)	1 (1.4)
Maghreb, Middle-East	94 (3.3)	16 (20.0)	6 (8.7)
Africa	52 (1.8)	0 (0)	3 (4.3)
Education			
< 1 universitary degree	444 (15.3)	68 (85.0)	55 (78.6)
1 universitary degree	357 (12.3)	6 (7.5)	5 (7.1)
2 universitary degrees	1054 (36.4)	4 (5.0)	7 (10.0)
3 universitary degrees	1037 (35.9)	0 (0)	3 (4.3)
Mensual outcome under poverty threshold (964€)			
Yes	513 (17.4)	62 (77.5)	60 (83.3)
Health insurance			
Yes	2425 (84.2)	11 (13.7)	18 (25.0)
Health insurance for the poor	168 (5.8)	42 (52.5)	19 (26.4)
None	287 (10.0)	27 (33.7)	35 (48.6)
Employment (declared or not)			
Yes	2206 (74.9)	25 (31.2)	11 (15.3)
No	739 (25.0)	55 (68.7)	61 (84.7)
Housing			
Stable housing	2458 (83.5)	13 (16.2)	40 (55.6)
Sheltered by friends	361 (12.3)	8 (10.0)	23 (31.9)
Other housing	126 (4.2)	59 (73.7)	9 (12.5)
Could be sheltered			
Yes	2626 (89.2)	34 (42.5)	48 (66.7)
Could have material help			
Yes	2506 (85.1)	28 (35.0)	31 (43.1)
Social support			
Yes	2594 (88.1)	45 (56.2)	52 (72.2)

**Table 2:** Sexual orientation, sexual risk behaviors and psychoactive substance uses in past 12 months in the three populations

	Checkpoint (*n* = 2945)	110 Les Halles (*n* = 80)	El Pasaje Latino (*n* = 72)
	N (%)	N (%)	N (%)
Sexual orientation			
Bisexual	425 (14.4)	2 (2.5)	11 (15.3)
Heterosexual	94 (3.2)	69 (86.2)	38 (52.8)
Homosexual	2309 (78.4)	0 (0)	15 (20.8)
Transgender	0 (0)	0 (0)	8 (11.1)
Don’t want to be define	54 (1.8)	1 (1.2)	
Missing	63 (2.1)	8 (10.0)	
Male sex partners			
Yes	2752 (93.4)	7 (8.7)	56 (77.8)
Female sex partners			
Yes	443 (15.0)	55 (68.7)	20 (27.8)
Transgender sex partners			
Yes	71 (2.4)	4 (5.0)	7 (9.7)
Total number of sex partners			
1	188 (6.6)	23 (37.7)	10 (14.8)
2 to 5	908 (31.7)	25 (40.9)	5 (7.3)
6 to 9	521 (18.2)	3 (4.9)	4 (5.9)
10 to 19	570 (19.9)	1 (1.6)	7 (10.3)
20 or more	534 (18.7)	3 (4.9)	40 (58.8)
missing	138 (4.8)	6 (9.8)	0 (0)
Unprotected intercourse			
Yes	1843 (64.3)	40 (65.6)	46 (63.9)
Transactional sex			
Yes	164 (5.7)	5 (8.2)	43 (59.7)
STI			
Yes	331 (11.2)	1 (1.2)	4 (5.6)
Having smoked drugs			
Yes	766 (26.0)	55 (68.7)	10 (13.9)
if yes, crack pipe sharing			
Yes	63 (8.2)	24 (43.6)	2 (20.0)
Having snorted drugs			
Yes	688 (23.4)	30 (37.5)	26 (36.1)
if yes, straw sharing			
Yes	268 (38.9)	6 (20.0)	15 (57.7)
Slam - substance use during sex			
Yes	35 (1.2)	14 (17.5)	0 (0)
if yes, needle sharing or other material			
Yes	4 (11.4)	7 (50.0)	0 (0)

**Table 3:** History of lifetime screening, past 12 months screening and HBV vaccination history in the three populations

	Checkpoint (*n* = 2945)	110 Les Halles (*n* = 80)	El Pasaje Latino (*n* = 72)
	N (%)	N (%)	N (%)
HIV screening			
Yes	2632 (89.4)	59 (73.7)	60 (83.3)
of which rapid tests	889 (33.8)	6 (10.2)	18 (30.0)
No	248 (8.4)	16 (20.0)	11 (15.3)
Missing	64 (2.2)	5 (6.2)	1 (1.4)
HIV screening past 12 months			
Yes	1868 (70.9)	29 (49.1)	22 (36.7)
No	595 (22.6)	28 (47.5)	38 (63.3)
Missing	170 (6.5)	2 (3.4)	0 (0)
Number of tests past 12 months			
One	1174 (50.0)	27 (87.1)	21 (36.1)
Two	391 (16.6)	2 (6.4)	28 (48.3)
≥ Three	303 (12.9)	0 (0)	9 (15.2)
Missing	170 (7.2)	1 (3.2)	0 (0)
HBV screening			
Yes	1471 (49.9)	50 (62.5)	48 (66.7)
No	1029 (34.9)	23 (28.7)	16 (22.2)
Missing	445 (15.1)	7 (8.7)	8 (11.1)
HBV screening past 12 months			
Yes	726 (49.3)	22 (27.5)	21 (29.1)
No	410 (27.9)	25 (31.2)	25 (34.7)
Missing	335 (22.8)	4 (5.0)	2 (2.8)
HCV screening			
Yes	1128 (38.3)	45 (56.2)	35 (48.6)
No	1096 (37.2)	23 (28.7)	26 (36.1)
Missing	721 (24.5)	12 (15.0)	11 (15.3)
HCV screening past 12 months			
Yes	610 (54.1)	24 (53.3)	22 (62.9)
No	273 (24.2)	19 (42.2)	13 (37.1)
Missing	245 (21.7)	2 (1.9)	0 (0)
HBV vaccination			
Yes, 3 doses	1216 (41.3)	29 (36.2)	26 (36.1)
history			
Not sure	650 (22.1)	14 (17.5)	16 (22.2)
No	410 (13.9)	27 (33.7)	15 (20.8)
Missing	668 (22.7)	10 (12.5)	15 (20.8)

Of the 2945 participants attending the CP, 47% were less than 29 years old (median age 30 years) with a high level of education and financial resources; 78.4% described themselves as MSM, 14.4% as bisexual and 3.2% heterosexual. The total number of sexual partners in the last 12 months was on average 10.5, 64.3% (*n* = 1838) had sex without a condom and 5.7% (164) had transactional sex in the previous year. Only 11.2% (*n* = 331) reported STIs in this period. A total of 41.2% (*n* = 1217) and 22.7% (*n* = 668) previously received a complete vaccine protocol against HBV and HAV, respectively. In terms of screening history, 50, 38 and 89% of MSM had already been tested for HBV, HCV and HIV respectively (49, 54 and 71% in the past 12 months).

Consenting PWIDs (*n* = 80) attending the 110-LH were mainly men (88.8%), with a median age of 42, a low education level (68.7% had a primary or middle school degree), a low level of financial resources (77.5% lived under the poverty threshold of 964€) and had no health-care insurance. Among them 47.4% were of French nationality and 36.2% were non European citizens, 73.7% had a previous HIV test, including 7.5% rapid tests, 56.3% HCV and 62.5% HBV previous screening.

The recruited SW/TGW (*n* = 72) attending PL were aged 40 or more (the median age was 37 years), mainly non-European citizens, with a low education level (61.1% had a primary or middle school degree), a low level of financial resources (83% lived under the poverty threshold) and with no health care insurance. Among them 22.2% were TGW, 38.9% male and 27.8% female, 52.8% were of French nationality. In terms of a screening history, 83.3% had already been tested for HIV, 25% of them by rapid tests, 62.9% for HCV and 66.7% for HBV.

The calculated acceptability rate for triple testing was of 98.5% (3662/3718) for MSM recruited in the CP and 14.9% (77/517) for the TGW/SW, in the PL center. A total of 82 triple tests were performed in PWIDs in the 110 LH center, but acceptability rate could not be calculated, since the number of users attending and number of proposals were not recorded.

A detailed flowchart is presented for the CP population (Fig. 1). Among this mostly MSM population, 3718 viral screening proposals were made per protocol, with 56 (1.5%) refusals. Among the main reasons for refusal were lack of time (*n* = 15), HIV/HBV/HCV status already known (*n* = 7). A total of 3662 triple tests were performed per protocol and 279 (7.6%) study questionnaires proposed at each testing were missing. Thus, the final analytical sample was of 2945 participants described at their first visit at the CP center. Among them, 385 volunteers had 2 visits, 65 had 3 visits, and nine had four visits.

### Testing results and linkage to care

A summary of the main results is presented in Table 4 for 2945 MSM participants. The results of rapid tests in MSM population refer to the first visit with a prevalence of 1.5% of HIV positive rapid tests (*n* = 44/2945). Of these, 9 participants were diagnosed at the primary infection stage (20.4%) and 19 at the recent infection stage (38.8%). Seven participants (0.24%) were positive for HBV only and 3/2945 (0.1%) for HCV only. Five participants were detected HIV positive at a later visit.

**Table 4:** Main results of the ANRS-CUBE intervention study

	Checkpoint	110 Les Halles	El Pasaje Latino
Period of inclusion	1/07/14 30/06/15	16/09/14 22/12/15	01/09/14 21/10/15
Targetted groups	MSM	PWIDs	TGW/SW
Number of users in 2013	4100	550	400
Number of expected participants	2500	110	81
Number of attendances	4042	Missing^a^	651^b^
Number of proposals	3718	82 ^c^	517
Number of refusals	56	23	440^d^
Number of participants tested per protocol	3662	82	77
Acceptability	98.5%	ND	14.9%
Numbers of participants who filled the questionnaire are their first attendance	2945	80	72
Number of new HIV positive participants	44 (1.5%)	4 (4.9%)	0 (0%)
Number of new HBV positive participants	7 (0.19%)	0 (0%)	0 (0%)
Number of new HCV positive participants	3 (0.8%)	5 (6.1%)	0 (0%)
Number of users eligible for HBV vaccination	1682	45	15
Number of HBV vaccination M0 (%)	301 (17.9%)	30 (66.7%)	8 (53.3%)
Number of HBV vaccination M1 (%)	203 (12.1%)	11 (24.4%)	4 (26.7%)
Number of HBV vaccination M6 (%)	97 (5.8%)	3 (6.7%)	0

MSM: men who have sex with men; PWIDs: people who inject drugs; TGW/SW: transgender women/sex workers

^a^The total number of attendances for this center was not available

^b^ The number of attendances was recorded on electronic database between 18/02/ and 21/10/2015

^c^ A large number of PWIDs had already been recently tested in anther CAARUD (harm reducing facilities for drug users) or in prison setting

^d^75% of the TGW/SWs declared being aware of their HIV serological status and HIV screening was not proposed or was refused

Among the 82 PWIDs tested at 110 LH center, an HIV infection was discovered in 4 patients (4.9%), three with a recent infection, a HCV infection in five (6.1%) patients (two were co-infected HIV-HCV). No newly diagnosed HBV infection was detected. Of the total of 82 PWIDs tested, two were already known as HIV positive, 6 HBV positive, and 20 HCV positive (two were co-infected for HBV and HCV and one for HIV-HCV).

Among the 77 TGW/SW tested at PL center, none were positive for any of the three viruses. Most of them (75%) declared being aware of their HIV status and testing was not proposed or refused.

Regarding HIV linkage of care, 47 among the 49 participants detected positive in the CP center, were addressed and reached health-care facilities and 44/47 were treated with antiretroviral therapy. Among these 44 participants, 30 initiated antiretroviral therapy within a months time. With regard to the HIV positive PWIDs, two out of four were referred to care facilities and were placed on antiretroviral drugs in less than 15 days. All participants discovered HBV and HCV positive in the CP center were successfully linked to care, as were the three mono-infected HCV patients in the 110LH center.

### Acceptability of a three-dose HBV vaccination

In terms of previous vaccination against HBV, the coverage was slightly higher in the MSM population (1216/2945, 41.3%) than in PWIDs (29/80, 36.2%) and TGW/SW (26/72, 36.1%). Among MSM participants, those with ages between 30 and 39, 40–49 and 50–59 presented the higher previous vaccination rates (60, 58.6 and 57.8%, respectively), compared to those aged between 18 and 29 (46%).

Data summarized in Table 4 shows the acceptability rates of HBV vaccination among eligible participants: 1682 MSM, 45 PWIDs and 15 TGW/SW. Among the eligible participants 301/1682 (17.9%), 30/45 (66.7%) and 8/15 (53.3%) accepted the first dose. Between half and two thirds of the participants having received their first dose, depending on the site returned for the second injection at M1 and even less for the last dose at M6 (97/301, 32.2% of MSM, 3/30, 10% of PWIDs and none of the TGW/SW).

## Discussion

To our knowledge, this is the first study that simultaneously evaluates the acceptability of a triple rapid screening (HIV/HCV/HBV) in three different specific target populations: MSM, PWIDs and TGW/SW, in a community setting. Taking into account the recent reported success of combined strategies of Test and Treat, TasP and PrEP in the reduction of new HIV infections, this kind of on-field, community targeted intervention should contribute to reinforce screening efforts. However, our study also highlights the challenges of putting into action such a combined strategy depending on the specificities of each target population.

Indeed, our study confirms the very high acceptability of screening by rapid tests in the MSM population (98.5%). The acceptability rate was weaker among SW/TGW (14.9%), but 75% (330/440) of refusals/tests not proposed were among individuals declaring being already aware of their HIV status. Moreover, despite a total of 82 viral screening tests performed in PWIDs in the 110 LH center, acceptability rate for this site could not be calculated, since the number of users attending and number of proposals were not recorded. These results show that for many MSM, rapid testing is incorporated into their practices (89.4% had already been tested for HIV) as a simple, socially-accepted prevention routine. With respect to TGW/SWs, peer support is important in terms of testing and treatment information and many of the tested TGW/SW had already been screened for HIV (83.3%). The difficulties for PWIDs to access to care have been already showed in a previous study, where PWIDs reported reasons linked to their own fears but also reasons that reflected reluctance from medical professionals [24]. However, our study allows us to explain some of the difficulties that harm reduction professionals have to face in their practice. Some of the main reason for the impossibility to record the number of proposals have been documented in the qualitative part of the study (not presented here), and were mainly explained by the workload of harm reduction professionals who are overwhelmed with daily requests of PWIDs more focused on basic needs such as finding a solution to avoid living outside, eating, washing clothes, and on addiction needs such as avoiding withdrawal by accessing opioid substitutive treatments, accessing sterile needles and crack pipes.

The following data also supports the results of our research on acceptability of triple testing. Point-of-care (POC) HIV/HBV/HCV/syphilis testing was previously assessed in two different settings and populations in two countries: in 375 sexually transmitted diseases clinic attendees in Mumbai, India and in 119 injection drug users in Montreal, Canada [25]. Feasibility was high: 86.1% in Mumbai and 92.4% in Montreal and authors concluded that a multiplex approach was preferred over conventional testing [25]. Bottero et al. in the OptiScreen III randomized control trial compared (1:1) standard serology-based with POC rapid testing and showed that POC screening improves rates of test results retrieval in a population of mainly African immigrants [17].. Other studies have reported low return rates after standard serology testing, pointing to a gain in viral-infection awareness when using rapid testing [26, 27].

Regarding community-based testing, the use of HIV rapid tests in developed countries was the subject of a general review, which included 44 studies, the majority conducted in the United States, most of them targeting MSM [28]. This meta-analysis confirmed varying levels of acceptance depending on the location of the rapid tests ranging from 9% in a bar to 95% in a mobile unit in a city setting, but satisfaction levels were high (91–99%). The rapid HIV test proposal has also been the subject of randomized studies in drug treatment centers in the United States, associated or not with counseling, and showed increased testing rates and results retrieval [29].

In our report the prevalence of HIV among the MSM/bisexual population was of 1.5% (*n* = 44), more than half (*n* = 28) of the discovered infections being recent or diagnosed at the primary stage. This underlines the importance of repeated frequent testing in this exposed population. The MSM population has already been identified as an “emergency in terms of public health”. With 18% of new HIV infections worldwide in 2017, it remains one of the most affected population in terms of HIV, HBV and to a growing extent, HCV [30]. Experts advocate HIV testing four times a year in MSM, with rapid tests, and recommend systematically combining HIV testing with other STIs including HBV to reduce the risk of co-transmission [31]. In France in 2018, 45% of new HIV infections were in MSM [9]. Even though the Paris region remains one of the most affected in terms of HIV prevalence, HIV new infections in MSM and bisexual men in this region only very recently begun to decrease with a of drop of 22% between 2015 and 2018, probably mainly due to the deployment of PrEP, combined with TasP. However certain populations like migrant MSM remain more difficult to reach [7, 9]. On-field testing interventions, similar to ours, combined to other strategies as self-testing, might represent efficient tools in order to increase screening opportunities, especially in a population otherwise less likely to access healthcare facilities.

Our study also highlights the deficit of HCV previous screening in MSM participants (38.4%). Seven participants (0.19%) were positive for HBV and 3 (0.8%) for HCV. These data are of concern in regards to the increasing incidence of HCV, mostly in HIV-positive MSM. A recent meta-analysis [32] indicates that the overall estimated incidence of HCV is 19-fold higher in HIV-positive compared to HIV negative MSM living in high-resource countries. Factors associated with an increased risk for HCV include behavioral factors (e.g. at risk sexual practices and drug use in sexual contexts with or without slamming), as well as biological characteristics (e.g. HIV co-infection and a recent history of STI) [14]. A French study also confirmed an increase in the incidence of acute HCV infection in HIV-infected patients, suggesting changes in sexual behavior in the recent years, especially in HIV-infected MSM and proposing repeated testing for HCV antibodies and re-enforcement of counseling [33].

In the PWIDs population, our data showed that included participants had relatively high rates of previous screening (73.7, 56.2 and 62.5% for HIV, HCV and HBV respectively). Few studies have examined the acceptability of the rapid test among PWIDs. Morano et al. found a similar acceptability of standard HCV and POC testing among clients of a mobile medical clinic, but participants accepting POC were more likely to be linked to care [34]. Hayes et al. found that the rapid HCV oral test was preferred to standard phlebotomy [35]. Another study evaluated POC testing in correlation with syringe-exchange programs (SEPs) and showed an acceptability rate of 85% among 413 participants [36]. In a recent review evaluating the effect of POC or dried blood spot (DBS) use on the uptake of HCV testing in high-risk populations, five of the six identified studies provided evidence that the introduction of DBS increased the number of tests, new diagnoses or both [35, 37]. Rapid testing efficacy in terms of acceptability and linkage to care needs to be further assessed, but associating it with other risk reducing interventions that have already proved efficient, as SEPs, might be a way to increase outreach.

In our study the rate of newly diagnosed HIV infections in PWIDs was high (4.9%), whereas that of HCV was of 6.1%, suggesting the benefits of this on-site screening intervention. In our study, the HCV incidence is lower compared to other studies conducted in France in the same time period with an 11% HCV incidence among PWIDs, which is quite high compared to other European countries such as the Netherlands or Switzerland which succeed to have very low levels of incidence due to strong and innovative harm reduction policies [38–40]. One of the critical result of our study regarding PWIDs is the high level of people who were newly HIV diagnosed, that has never been documented before but which confirms a critical trend observed among PWIDs in France in regards to the increase of at-risk practices these last years in this population [41]. Notably, many of the 82 tested PWIDs had already been screened in another community center or during incarceration, suggesting the importance of repeated testing. More generally, according to the ANRS-Coquelicot survey from 2011 to 2013 French HIV and HCV seroprevalences among PWIDs were respectively 13 and 64% [11]. Higher HIV rates were reported in Paris and northern suburbs (14 and 21% respectively) in older users (age over 35 years) and HCV rates were also estimated as being higher than in the other geographical areas, regardless of age [11]. Paris region has several particularities regarding PWIDs. Among them are an older age, as reflected also by our study population and several conditions regarding higher risk-taking (sharing injection material, extreme precarity, difficulties to obtain sterile syringes and injection in public spaces), which are known factors for HIV and HCV acquisition [11].

Among TGW/SW that attended the PL none of the tested participants were found positive for any of the three viruses. In those tested, the intervention was not perceived as stigmatizing and as showed above the majority of the TGW/SW included or that refused testing had already been screened for HIV (83.3 and 75%, respectively), thanks to support paths adapted to the specificities of the Spanish-speaking public. Most of the volunteers were of non-European origin; all represented vulnerable populations and had regular follow-up in the community center.

In accordance with our data, in France, a high proportion of migrant trans-women originate from countries of South America, where HIV prevalence in this group is high (18–38%) [42]. Regarding HIV prevalence in the TGW/SW population, most data concern trans-women. The results of a meta-analysis from 2000 to 2011 including 39 studies in 15 different countries, showed a 19% prevalence among trans women [43]. According to UNAIDS, globally it is estimated that transgender women are 13 times more likely to acquire HIV than other adults of reproductive age [30].

In terms of HBV vaccination, the coverage was similar in the MSM population, compared to PWIDs and SW/TGW (41.3%, compared to 36.2 and 36.1% respectively). Regarding HBV vaccination among PWIDs, the level of vaccination is lower compared to another study done at the same period where HBV vaccination reached 60%, but it might be explained by the fact that our study recorded the number of doses, which allows more reliable data [13]. Previous HBV vaccination was lowest among MSM aged between 18 and 29 (46% compared to 60% in those aged 30–39), knowing that in France the hepatitis B vaccine was licensed in 1982,universal infant vaccination recommended since 1994 but scholar vaccination in teenagers has been discontinued in 1998. Also, the vaccination refusal rate was surprisingly high among MSM (82.1%) even though this specific population is targeted by different prevention strategies and is generally better informed and less precarious than the other study groups. This emphasizes the need to continuously reinforce vaccination campaigns, especially in youngsters and in at-risk groups. Compliance with a three-dose vaccination schedule over 6 months was difficult in all study groups. Potential benefits of accelerated vaccination programs in these specific populations need to be better assessed and might represent a way to improve adherence.

In the qualitative part of the study, we also observed clear resistances of caregivers to propose HBV vaccination systematically and some confusion concerning the use of the HBsAg screening test for the vaccination proposal. Some actions have already demonstrated their efficiency for increasing vaccination rates, for example in PWIDs: providing free vaccination in specialized centers, increasing awareness among healthcare workers regarding HBV and its prevention, implementing an accelerated HBV vaccination schedule, offering financial incentives to PWIDs to enhance their adherence to the vaccine schedule and providing greater access to hepatitis testing [44–47]. In MSM, access to PrEP, coupled with counseling and systematic STD, including HBV screening, may also represent an important step in order to improve vaccination rates.

Some limitations of our study need to be addressed. First, this was a not randomized triple-center pilot study and data does not allow extrapolation to other testing centers and other key populations. Moreover, all participating centers where located in Paris, a city with particular characteristics regarding HIV/HBV and HCV epidemics compared to most other French regions. Several factors were center-specific, for example previous expertise with screening. Some professionals managing PWIDs perceived the rapid testing proposal as disrupting their daily work in the management of social insecurity and psychiatric co-morbidities of PWIDs, which did not allow to implement as well as expected the study procedures. Even though during the study period, the professionals were able to improve structure organization in order to make implementing the intervention possible, staff involvement was variable at each center and between centers and also in regard to each intervention (viral testing vs vaccination). Second, the number of participants and infected individuals may have decreased power to detect certain differences between the three studied populations, mostly in terms of linkage-to-care. Also we did not calculate the correlation of finance or education with acceptability rates. Educational attainment has already been correlated with uptake of HIV testing [48, 49]. Third, in one center, addressing PWIDs, we could not determine the level of acceptability. Finally, not all rapid tests were approved for routine use in France, and testing was conducted in a research setting.

## Conclusion

Our results showed that the strategy of a triple rapid HIV-HCV-HBV screening had a high level of acceptability among MSM, while the implementation of the strategy seemed more complicated in the 110 LH center, where support is required to extend medical practices in screening and vaccination and to frame the announcement of positive tests. The immediate proposal for HBV vaccination appeared to be poorly accepted by MSM and full compliance with a 6-months three-dose vaccination schedule was an unmet challenge in all study groups. Our results show the important role of community centers for reaching specific populations living with discrimination under difficult social conditions and far from the health-care system. Testing and re-testing for diagnosis of HIV, HBV, and HCV infection remain the pillars for access to both treatment and prevention services, and crucial for an effective HIV and viral hepatitis epidemic response.

## Data Availability

The datasets used and/or analysed during the current study available from the corresponding author on reasonable request.

## Abbreviations

110LH: 110 Les Halles
ANRS: France Recherche Nord & Sud Sida-HIV Hépatites
ANSM: Agence Nationale pour la Sécurité du Médicament
CI: confidence intervals
CNIL: National Commission of Informatics and Liberties
CP: Checkpoint Paris
CSAPA: Center of Care, Support and Prevention in Addiction
DBS: dried blood spot
HBs Ag: Hepatitis B surface antigen
HBV: Chronic hepatitis B virus infection
HCV: chronic hepatitis C virus
HIV: Human immunodeficiency virus
MSM: men who have sex with men
PL: El Pasaje Lation
POC: point of care
PrEP: pre-exposure prophylaxis
PWIDs: people who inject drugs
SEPs: syringe-exchange programmes
STI: sexually transmitted infection
SW: sex workers (SW)
TasP: treatment-as-prevention
TG: transgender
TGW: transgender women
UNAIDS: Joint United Nations Programme on HIV and AIDS
WHO: World Health Organisation
